# Nowcasting by Bayesian Smoothing: A flexible, generalizable model for real-time epidemic tracking

**DOI:** 10.1371/journal.pcbi.1007735

**Published:** 2020-04-06

**Authors:** Sarah F. McGough, Michael A. Johansson, Marc Lipsitch, Nicolas A. Menzies

**Affiliations:** 1 Department of Global Health and Population, Harvard T.H. Chan School of Public Health, Harvard University, Boston, Massachusetts, United States of America; 2 Division of Vector-Borne Diseases, Centers for Disease Control and Prevention, San Juan, Puerto Rico; 3 Center for Communicable Disease Dynamics, Department of Epidemiology, Harvard T.H. Chan School of Public Health, Boston, Massachusetts, United States of America; 4 Department of Immunology and Infectious Diseases, Harvard T.H. Chan School of Public Health, Boston, Massachusetts, United States of America; University of Virginia, UNITED STATES

## Abstract

Achieving accurate, real-time estimates of disease activity is challenged by delays in case reporting. “Nowcast” approaches attempt to estimate the complete case counts for a given reporting date, using a time series of case reports that is known to be incomplete due to reporting delays. Modeling the reporting delay distribution is a common feature of nowcast approaches. However, many nowcast approaches ignore a crucial feature of infectious disease transmission—that future cases are intrinsically linked to past reported cases—and are optimized to one or two applications, which may limit generalizability. Here, we present a Bayesian approach, NobBS (Nowcasting by Bayesian Smoothing) capable of producing smooth and accurate nowcasts in multiple disease settings. We test NobBS on dengue in Puerto Rico and influenza-like illness (ILI) in the United States to examine performance and robustness across settings exhibiting a range of common reporting delay characteristics (from stable to time-varying), and compare this approach with a published nowcasting software package while investigating the features of each approach that contribute to good or poor performance. We show that introducing a temporal relationship between cases considerably improves performance when the reporting delay distribution is time-varying, and we identify trade-offs in the role of moving windows to accurately capture changes in the delay. We present software implementing this new approach (R package “NobBS”) for widespread application and provide practical guidance on implementation.

## Author summary

Achieving accurate, real-time estimates of disease activity is challenged by delays in case reporting. However, approaches that seek to estimate cases in spite of reporting delays often do not consider the temporal relationship between cases during an outbreak, nor do they identify characteristics of robust approaches that generalize to a wide range of surveillance contexts with very different reporting delays. Here, we present a smooth Bayesian nowcasting approach that produces accurate estimates that capture the time evolution of the epidemic curve and outperform a previously developed approach. We assess the performance for two diseases and show that relating cases between sequential time points contributes to NobBS’s performance and robustness across surveillance settings.

## Introduction

Effective public health action relies on surveillance that is timely and accurate, especially in disease outbreaks[1,2]. Specifically, surveillance provides the information required to assess risks, prioritize and allocate resources to public health threats, deploy and discontinue interventions to interrupt transmission, and monitor the impact of those interventions. Ideally, disease surveillance systems should closely track the often fast-changing circumstances of outbreaks, distinguishing true changes in the dynamics from artifacts of reporting.

Despite the importance of timely surveillance data, substantial challenges exist to collect and report case information in real time. Multiple features of the disease and surveillance system contribute to reporting delays, including: delays in symptoms onset after infection; delays in medical care-seeking after onset; delays in providers obtaining and reporting diagnostic information; level of awareness of disease activity influencing care-seeking and reporting; and system-level processing delays, a result of complex and multi-tiered disease reporting and communication systems interacting at multiple administrative levels[3]. Reporting delays can be further exacerbated in resource-constrained settings. As a consequence, surveillance data are typically not complete until weeks or months after infections have actually occurred, providing an incomplete picture of current disease activity.

Nowcasting, or “predicting the present,” is an approach to mitigate the impact of reporting delays. With origins in the insurance claims and actuarial literature[4,5], nowcast models aim to estimate the number of occurred-but-not-yet-reported events (e.g. insurance claims, disease cases) at any given time based on an incomplete set of reports. In public health settings, nowcasting approaches have been explored for AIDS in the 1980s and 1990s[6–8] as a consequence of the long incubation period from HIV infection until development of AIDS. More recently, nowcasting has been applied to infectious disease outbreaks such as foodborne illness outbreaks[9,10]. These studies draw principally on survival analysis and actuarial techniques to model the reporting delay and draw inferences based on historical patterns. A majority of studies have strictly focused on modeling the reporting delay distribution—a legacy of the actuarial techniques giving rise to many of these approaches—and generally neglect a key feature of outbreaks: that future cases are intrinsically linked to past reported cases, a fact that creates potentially strong autocorrelation in the true number of cases over short time intervals. In other words, the infectious disease transmission process provides an additional signal of the number of cases to be expected in the near future that has not been included in common methods such as the reverse-time hazard model[11,12] and the chain ladder method[13], which model the probability of delays in case reporting and consider the number of cases at successive time points as independent. However, proposals to extend the chain ladder approach to state-space models that account for temporal relationships in reporting have existed in the literature since the development of these techniques[13–15] and have been applied in at least one infectious disease context[16].

The development of nowcast models is promising for disease surveillance, but equally important is the need to identify and scrutinize the mechanisms by which these approaches are successful in accurately predicting true case reports and, on the other hand, underperform. For example, it is critical to demonstrate performance in a diversity of settings as a majority of disease nowcast models to date have largely focused on performance in specific applications, but not the common challenges that exist across many different diseases. To wit, in this investigation we find that nowcasting is especially challenging when the proportion of cases reported the week they occur (delay 0) is low and reporting delays are highly variable; we know of no investigations that specifically identify models that perform well in these commonly occurring circumstances. As a result, the characteristics of robust and broadly-applicable models are difficult to identify. Additionally, several previous models have largely focused on providing point estimates of the number of cases. Point estimates may be helpful, but quantifying the uncertainty in those estimates is even more important in the context of infectious disease outbreaks because uncertainty is intrinsic, and accounting for plausible outcomes apart from the point prediction is critical.

Here, we implement a relatively disease-agnostic nowcast method in multiple real-world and simulated disease reporting settings, and unpack the features of model structure and implementation that contribute to its performance. We introduce Nowcasting by Bayesian Smoothing (NobBS), a simple and flexible generalized Bayesian model and associated software package for nowcasting infectious diseases. We demonstrate the robustness of this approach in two very different disease surveillance contexts and identify the conditions that favor its application, especially when the reporting delay distribution is time-varying. Specifically, NobBS allows for both uncertainty in the delay distribution and the time evolution of the epidemic curve, producing smooth, time-correlated estimates of cases. We demonstrate that NobBS performs well for weekly nowcasts of (1) dengue cases in Puerto Rico and (2) influenza-like illness (ILI) cases in the United States, requiring no disease-specific parameterization despite the two pathogens being very different (vector-borne vs. directly transmitted) and exhibiting substantially different reporting delays. Lastly, we test NobBS against a previously developed Bayesian nowcast method for surveillance data[9] and find that NobBS outperforms this established method for both diseases and multiple time periods. In particular, we show that while point estimates of the models are similar when time-to-report distributions are relatively fixed over time, NobBS improves the estimation of uncertainty and accommodates temporal variation in delay probabilities. Our investigation aims to provide deeper insights into these different nowcast approaches and describe the practical implementation of NobBS. We present an R package, “NobBS,” as a tool to complement both routine public health surveillance as well as forecasting efforts.

## Results

We developed a Bayesian approach to nowcast total case numbers using incomplete, time-stamped reported case data based on an estimated delay distribution, intrinsic autocorrelation from the transmission process, and historical case data. Generally, the approach learns from historical information on cases reported at multiple delays (e.g. no delay, 1-week delay, 2-week delay, etc.) from the week of case onset to estimate the reporting delay probability at each delay and the relationship between case counts from week-to-week, and uses this relationship to predict the number of not-yet-reported cases in the present using a log-linear model for the number of cases at each week *t* and each delay *d*. We tested this approach, NobBS, using two different infectious disease surveillance data sources: dengue surveillance in Puerto Rico, and national notifications of influenza-like illness (ILI) in the United States. Using all of the available data on case reporting delays up to the point of prediction, weekly dengue nowcasts were estimated for the time period December 23, 1991 through November 29, 2010 (989 weeks), and weekly ILI nowcasts were produced over the period June 30, 2014 through September 25, 2017 (170 weeks). For comparison, we generated weekly nowcasts over the same periods using an existing Bayesian approach by Höhle and der Heiden (2014), here referred to as the HH approach[9]. Briefly, this approach frames the nowcast problem in the context of survival modeling (specifically, a reverse-time discrete hazard model) and estimates the delay distribution to obtain the total number of cases each week at each time *t*. We chose this approach as a comparator because it is the most established and implementable approach, with an existing R package and the most cited in disease surveillance contexts[9]. To leverage a large amount of historical data to fit the nowcast model while also having a large window over which to assess nowcasts, we used a 104-week (approximately 2-y) moving window dengue and a 27-week (approximately 6-mo) moving window for ILI and tested the sensitivity to window size to inform model implementation. Our primary outcome metric to assess nowcast performance was the logarithmic score, a proper score that evaluates the probability assigned to the observed outcome rather than error associated with a point prediction. For purposes of discussion, we reported the exponentiated form of the mean logarithmic score (the geometric mean of the assigned probabilities) to provide a metric on the scale of 0 (no certainty of the outcome) to 1 (complete certainty of the outcome). In addition, we estimated other metrics describing the performance of point estimates (mean absolute error (MAE), root mean square error (RMSE), and relative root mean square error (rRMSE)) and the 95% prediction interval (PI) coverage, and of these, focus on comparing the rRMSE and 95% PI coverage across approaches.

### Performance in forecasting weekly dengue and influenza incidence

**Fig 1:**
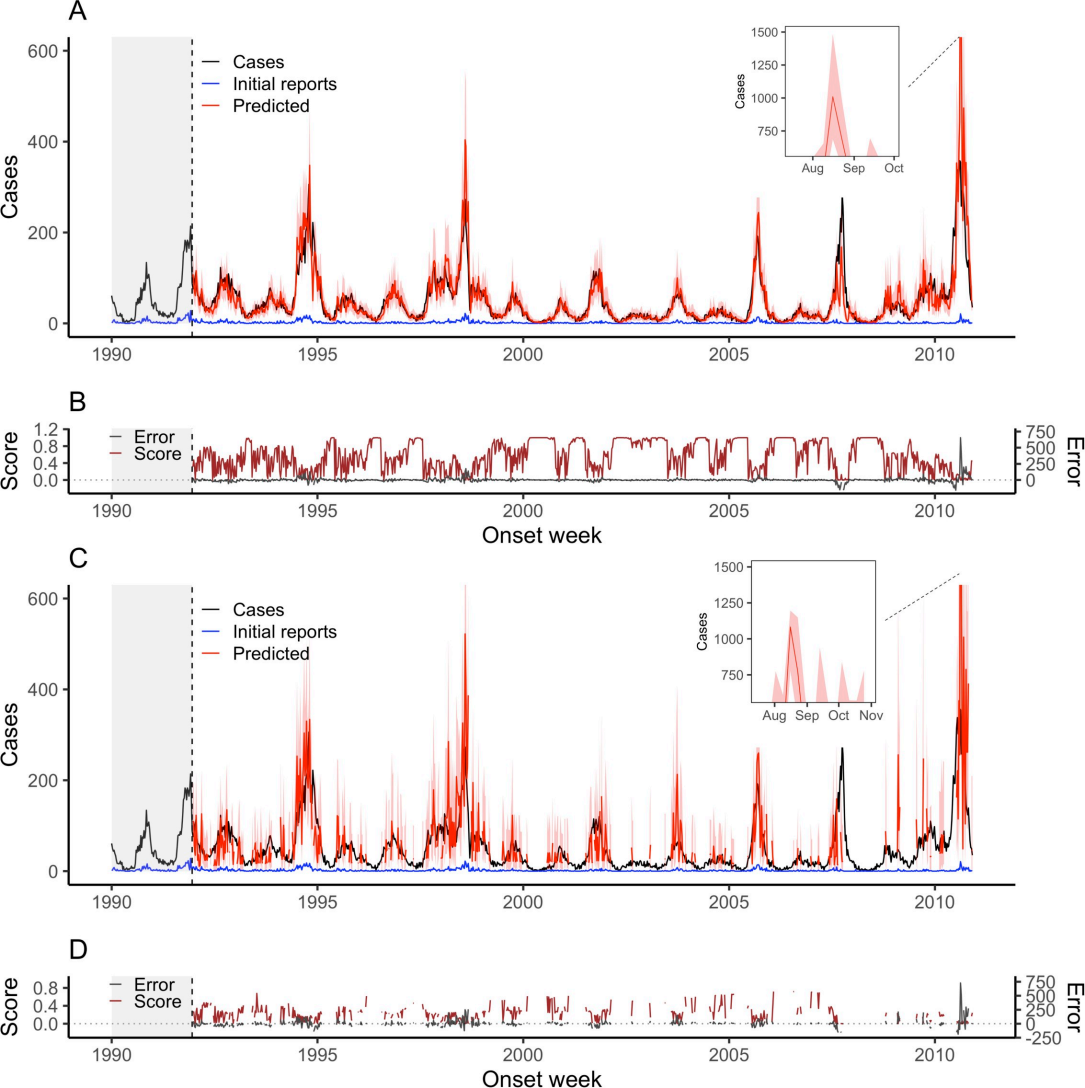
Weekly dengue fever nowcasts for December 23, 1991 through November 29, 2010 using a 2-year moving window. (A) Weekly NobBS nowcasts along with (B) point estimate and uncertainty accuracy, as measured by the score and the prediction error, are compared to (C) weekly nowcasts by the HH approach with (D) corresponding scores and prediction errors. For nowcasting, the number of newly-reported cases each week (blue line) are the only data available in real-time for that week, and help inform the estimate of the total number of cases that will be eventually reported (red line), shown with 95% prediction intervals (pink bands). The true number of cases eventually reported (black line) is known only in hindsight and is the nowcast target. Historical information on reporting is available within a 104-week moving window (grey shade) and used to make nowcasts. The score (brown line) and the difference between the true and mean estimated number of cases (grey line) are shown as a function of time.

**Fig 2:**
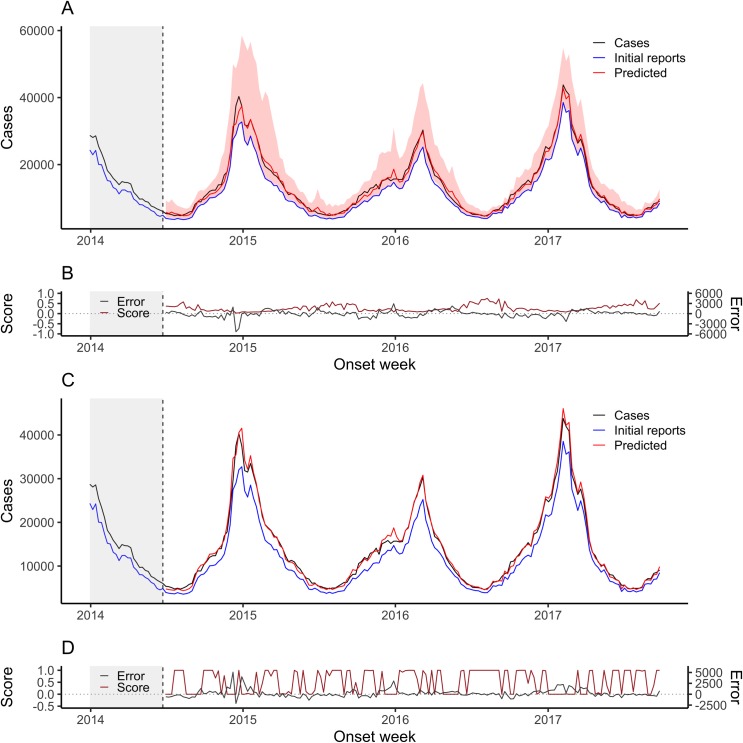
Weekly ILI nowcasts for June 30, 2014 through September 25, 2017 using a 6-month moving window. (A) Weekly NobBS nowcasts along with (B) point estimate and uncertainty accuracy, as measured by the score and the prediction error, are compared to (C) weekly nowcasts by the HH approach with (D) corresponding scores and prediction errors. For nowcasting, the number of newly-reported cases each week (blue line) are the only data available in real-time for that week, and help inform the estimate of the total number of cases that will be eventually reported (red line), shown with 95% prediction intervals (pink bands). For the HH approach, the 95% prediction intervals are very narrow and are thus difficult to see. The true number of cases eventually reported (black line) is known only in hindsight and is the nowcast target. Historical information on reporting is available within a 27-week moving window (grey shade) and used to make nowcasts. The score (brown line) and the difference between the true and mean estimated number of cases (grey line) are shown as a function of time.

Figs 1 and 2 show weekly dengue and ILI nowcasts for NobBS and the HH approach over multiple seasons for both diseases. Table 1 summarizes the point and probability-based accuracy metrics for each, where higher accuracy is indicated by higher average scores, lower MAE, RMSE, and rRMSE, and lower distance from 0.95 for the 95% PI coverage. Because the NobBS model accounts for both under-reporting and the autocorrelated progression of transmission across successive weeks, it makes predictions even in weeks when there are no cases reported for the week. Conversely, the HH model does not make nowcasts for weeks in which there are no initial case reports (common in the dengue Puerto Rico data), hence the nowcasts in Figs 1C and 2C appear as discontinuous lines. To compare models despite these differences, we report accuracy metrics between NobBS and the HH approach for both (1) the full time series of the data and (2) weeks when at least one case was reported in the first week, i.e. the subset of weeks for which both models could make predictions (Table 1). We also computed error metrics for the HH model for the full time series by assigning point estimates of 0 cases for nowcasts in weeks without predictions.

**Table 1:** Performance measures for each nowcast approach by disease (mean % reported with no delay, computed across the complete time period* of reports).

Disease	Model	Period	% of weeks predicted	Average Score	MAE	RMSE	rRMSE	95% PI coverage
Dengue	NobBS	Full time period*	100%	0.349	16	37.6	0.600	0.87
(4%)		Weeks in which at least 1 case was reported in the first week	--	0.274	21	46.6	0.464	0.85
	HH (ref. 9)	Full time period*	55%	--	32	57.4	1.14	--
		Weeks in which at least 1 case was reported in the first week	--	0.161	37	68.1	1.24	0.90
Influenza	NobBS	Full time period*	100%	0.218	693	987.8	0.074	1.00
(82%)								
	HH (ref. 9)	Full time period*	100%	0.017	609	916.2	0.062	0.00

*Full time period for: dengue fever (12/23/1991-11/29/2010) and ILI (6/30/2014-9/25/2017)

The HH approach made predictions in only 55% of weeks in the dengue time series (Table 1). In this subset of weeks, the NobBS approach achieved relatively smooth and accurate tracking of the dengue time series (rRMSE = 0.464, average score = 0.274) despite low proportions of cases reported on the week of onset (Fig 1A and 1B). The 95% PI coverage was 0.85, indicating that the 95% PI included the true number of cases for 85% of the nowcasts. In comparison, the HH approach produced substantially less accurate point estimates and slightly broader uncertainty intervals (rRMSE = 1.24, average score = 0.161, 95% PI coverage = 0.90) with greater fluctuation in nowcasts from week-to-week (Fig 1C and 1D). On average, the HH approach led to more biased estimates and systematically overpredicted the number of cases for both ILI and dengue compared to NobBS. Because many weeks in the dengue data were low incidence, assigning a prediction of 0 to the HH approach’s missing nowcasts improved its rRMSE to 1.14 in the full time series compared to 1.24 for the subset over which nowcasts were generated from the model, though NobBS still surpassed the HH model’s accuracy on this and all other metrics (Table 1).

Nowcast point estimates tracked the ILI time series well for both approaches, though point estimates had greater error by all measures for the NobBS approach (NobBS rRMSE = 0.074 vs. HH rRMSE = 0.062; Table 1). However, the NobBS approach produced considerably wider prediction intervals (Figs 1C and 2C) resulting in both higher scores (NobBS average score = 0.218 vs. HH average score = 0.017) and 100% coverage by the 95% prediction intervals compared to 0% coverage for the HH (Table 1). We observed that at multiple deciles of the prediction interval, the interval provided conservative coverage, but that the calibration of narrower intervals was better (S1 Fig).

To assess the degree of autocorrelation and related smoothness in the NobBS predictions, we calculated the 1-week lagged autocorrelation of predictions (ρ_a_) and compared this to the 1-week lagged autocorrelation of cases (ρ_c_). In addition, we computed metrics reflecting the accuracy of the approaches in capturing the *change* in cases from week-to-week: the mean absolute error of the change (MAEΔ) and the RMSE of the change (RMSEΔ) (Table 2). The magnitude of change was much larger for the ILI data than dengue data, with average absolute value change of 1,312.6 cases/week versus 9.8 cases/week, yet both showed high autocorrelation (ρ_c_ = 0.958 for dengue and ρ_c_ = 0.972 for ILI). Comparing the full time series, the nowcasts produced by NobBS exhibited high autocorrelation for both diseases (ρ_a_ = 0.876 for dengue, 0.973 for ILI) while the HH approach yielded lower autocorrelation for dengue nowcasts, comparatively (ρ_a_ = 0.631 for dengue, 0.970 for ILI). For dengue, over the weeks in which at least 1 case was initially reported, the NobBS approach achieved both lower mean absolute difference between predicted and observed changes in cases (NobBS MAEΔ = 23 vs. HH MAEΔ = 50) and lower RMSE of the change (NobBS RMSEΔ = 35.8 vs. HH RMSEΔ = 64.6). In addition, NobBS outperformed the HH approach over the full time series of dengue cases (Table 2). For ILI, however, the metrics for the weekly change were similar for the two approaches (Table 2).

**Table 2:** Performance measures for estimates of the change in disease incidence from the previous week.

Model		Dengue (mean cases/week = 48)				Influenza (mean cases/week = 14,000)			
	Period	MAEΔ	RMSEΔ	ρ_a_	ρ_c_	MAEΔ	RMSEΔ	ρ_a_	ρ_c_
NobBS	Full time period*	17	35.8	0.876	0.958	669	1027.1	0.973	0.972
	Weeks in which at least 1 case was reported in the first week	23	45.2	--	--	--	--	--	
HH (ref. 9)	Full time period*	34	64.6	0.631	0.958	612	1004.2	0.970	0.972
	Weeks in which at least 1 case was reported in the first week	50	88.2	--	--	--	--	--	

*Full time period for: dengue fever (12/23/1991-11/29/2010) and ILI (6/30/2014-9/25/2017)

### Reporting delays impact nowcast performance

The delay distributions between the reporting systems are strikingly different (Figs 1 and 2, S2 Fig). In the case of the dengue surveillance system, which includes specimen collection and laboratory testing, only approximately 4% of cases were processed during the week of onset, on average. In contrast, the U.S. Outpatient Influenza-like Illness (ILI) Surveillance Network (ILINet) captures only syndromic data reported electronically, with over 80% of ILI cases reported, on average, the same week they present (i.e. with no delay). Overall, we observed that the accuracy of nowcast point estimates (rRMSE) was higher for the ILI data compared to dengue, which may be related to the high proportion of cases reported with 0-weeks delay in these data. Large weekly absolute changes in the number of initial case reports also appeared to be related to increased error, particularly for dengue, which had high fluctuations in the number of initial reports over time (S1 Table, S3 Fig). Note that because of the difference in predictive distribution bin widths based on the number of cases that accrue for influenza vs. dengue (*Materials & Methods*), average scores are not comparable across diseases.

### NobBS improves nowcasting with varying reporting delays

Dengue and ILI also exhibit differences in the *trends* of reporting delay probabilities *over time*, and we observed strikingly different comparative performance of the two nowcast approaches in these disease settings, namely that the HH approach performed comparably to NobBS for ILI but not for dengue. In the dengue data, we observe a noisier, more time-varying probability of reporting for cases, with more extreme fluctuations in the proportion of initial reports compared to ILI cases, which show more constant (tighter ranges of) reporting probabilities from week-to-week (S4 Fig). Independent of the initial proportion of cases reported (high vs. low), we hypothesized that these trends (relatively constant vs. time-varying) are particularly impactful on the performance of the nowcast, and that relatively constant reporting probabilities, as seen in the ILI data, may be linked to the higher accuracy of these predictions.

To test the robustness of the model, we simulated ILI data using the final counts from the true dataset, but imposing a time-varying delay distribution; specifically, with faster initial reporting during weeks of high incidence to reflect heightened awareness of an outbreak and increased urgency of reporting (described in *Materials and Methods*). Using these simulated data, we found that NobBS was relatively robust to changes in reporting delays (S5 Fig, S2 Table). In the context of stable reporting delays (original ILI data), NobBS performed comparably to the HH model (Fig 2, Table 1). However, in the presence of simulated time-varying reporting delays, NobBS outperformed the HH in terms of confidence (NobBS average score = 0.06 vs. HH average score ≈ 0), point estimates (NobBS rRMSE = 0.302 vs. HH rRMSE = 0.621), and accuracy of the predicted change (S3 Table). Such variations in the delay distribution are a reality in many epidemics[17].

Time-varying delays may be explicitly modeled in the HH approach, by pre-specifying the temporal change-points at which the delay distribution is expected to change–information that is rarely known in real-time. We therefore compared NobBS to the HH approach without explicitly modeling a time-varying delay distribution in either method. In the case of ILI and dengue data, we had no reason to believe or anticipate a systematic change in reporting over the time period covered.

### Performance by year

The performance of both approaches in nowcasting ILI was relatively consistent by year across accuracy measures, but there were fluctuations in the year-to-year performance of dengue nowcasts (Table 3). Average scores of the dengue nowcasts (from both approaches) tended to be high in years that experienced a very low number of dengue cases (e.g. 2000, 2002, 2004, 2006), and therefore the performance of both approaches declined during higher incidence years. For dengue, the NobBS model was particularly effective at identifying periods of low incidence, with high probabilities assigned to the lowest outcome bin (0–25 cases, details in *Materials and Methods*) when the number of cases eventually reported was low (S6 Fig). On the other hand, during periods of high dengue activity, lower probabilities were assigned to the correct bin, reflecting greater uncertainty. Looking specifically at higher incidence time periods (Epidemiologic Weeks 40 through 20 of the following year for ILI, and all weeks in which the number of cases exceeded 50 for dengue), both nowcast approaches were less accurate and more uncertain than during other time periods (S4 Table). Overall, NobBS outperformed the benchmark approach on all performance measures for each year and most higher incidence time periods (Table 3; S4 Table).

**Table 3:** Annual performance measures for each nowcast model, by disease. All predicted weeks for each model are compared.

Disease			NobBS				HH (ref. 9)			
	Year	Cases	MAE	rRMSE	RMSE	Average Score	MAE	rRMSE	RMSE	Average Score
Dengue	1992	3,570	15	0.271	19.7	0.262	27	0.473	33.2	0.154
	1993	2,044	10	0.325	13.0	0.436	20	0.559	23.5	0.237
	1994	5,455	29	0.356	45.9	0.171	50	0.690	63.3	0.108
	1995	2,075	13	0.450	16.2	0.330	28	1.035	38.4	0.178
** **	1996	1,856	8	0.520	11.0	0.472	17	0.617	21.9	0.270
	1997	2,413	12	0.375	16.2	0.402	20	0.625	26.7	0.228
	1998	5,334	33	0.448	47.8	0.129	65	0.801	89.9	0.072
	1999	1,823	9	0.389	11.9	0.493	18	0.897	23.5	0.250
	2000	766	4	0.359	6.1	0.720	17	2.225	20.2	0.304
	2001	2,274	11	0.487	16.6	0.437	26	0.492	37.7	0.189
	2002	821	5	0.522	5.7	0.834	16	1.101	23.0	0.352
	2003	1,422	6	0.471	9.5	0.590	32	1.412	47.5	0.193
	2004	911	6	0.599	7.2	0.610	13	2.088	17.0	0.368
** **	2005	2,543	14	0.998	21.4	0.407	32	1.150	42.0	0.178
	2006	734	4	0.891	6.3	0.770	13	1.211	15.8	0.395
	2007	3,290	30	0.675	55.4	0.102	55	0.632	93.6	0.066
** **	2008	843	8	1.032	12.8	0.629	38	4.145	50.7	0.191
	2009	2,448	19	0.667	26.7	0.225	57	2.405	81.9	0.092
	2010	6,820	71	0.583	132.4	0.055	121	0.854	198.7	0.041
Influenza	2014	726,312	1052	0.085	1565.9	0.188	958	0.091	1482.0	0.004
	2015	679,850	685	0.086	890.3	0.203	624	0.069	848.0	0.019
	2016	704,020	696	0.072	861.8	0.224	376	0.043	480.1	0.063
	2017	632,353	551	0.046	712.9	0.258	659	0.047	934.2	0.008

Both approaches had their lowest accuracy on three high incidence dengue seasons: 1994, 2007, and 2010 (Table 3; S4 Table; Fig 1). The average annual scores for these years range between 0.041 and 0.170 across the NobBS and benchmark approaches, and between 0.000 and 0.081 for the high-incidence time periods specifically (Table 3; S4 Table), falling clearly below the rest of the years and higher incidence time periods in performance. These scores not only reflect unusually poor point estimate predictions as judged by rRMSE, but also the finding that the predictive distribution for weeks in these years for both approaches rarely included the true value of interest (a consequence of dramatic over- or underestimates during times of high disease activity).

### Moving window sizes

We initially used moving windows of 104 weeks for dengue (a longer time series) and 27 weeks for ILI (a shorter time series) to leverage a large number of historical training weeks to train nowcast estimates. Moving windows allow for stable estimation of the recent delay distribution as information from very old and potentially less relevant weeks are forgotten. The size of the moving window reflects how quickly and smoothly changes in the data should be realized by the model: longer moving windows tend to produce smoother estimates, but the model may be less sensitive to abrupt changes in the data (e.g. changes in how quickly cases are reported during an outbreak) or shorter-interval secular trends, e.g. seasonality.

While we chose long moving windows for both diseases to capitalize on data availability, these considerations may affect the choice of moving window size and nowcast performance, depending on the data. In light of this, we experimented with moving windows of different lengths on the dengue data to assess the impact on nowcast performance. We tested moving windows of 5, 12, and 27 weeks (approx. 6 months) on the dengue data and, for each moving window, generated a set of nowcasts for December 1991-November 2010 in order to compare performance measures across all moving window sizes (5, 12, 27, and 104 weeks). A 5-week moving window produced substantially lower accuracy nowcasts (rRMSE = 7.381) with several steep case overestimates in 2007–08 and 2010 (S7A Fig). However, accuracy metrics for moving windows of 12 weeks or longer were similar to those using the full 104 week window (range in rRMSE: 0.6–0.655; average score: 0.35–0.37) (S5 Table; S7 Fig). While shorter moving windows often produced more accurate estimates of the reporting delay probability (S8A Fig), the estimated variance of the random walk process varied dramatically from week-to-week (S8B Fig) resulting in more dramatic overestimates at certain periods of extreme time-varying delays, suggesting a trade-off between delay estimation accuracy and more stable estimates of weekly cases.

## Discussion

We introduce an approach for Bayesian nowcasting, NobBS, and demonstrate its application in two disease contexts with different reporting systems, investigating the conditions that contribute to successful implementation. We show that NobBS outperforms an existing method in terms of point estimate (reduced RMSE, lower bias) and probabilistic (higher logarithmic score) predictive performance. In particular, NobBS performs well even when the delays in case reporting change over time. We further demonstrate an important trade-off related to moving window sizes for delay distribution estimates; short windows improve the real-time characterization of the delay distribution but are susceptible to over-estimating that variability, potentially decreasing nowcast accuracy. Lacking any disease-specific parameterization, and relying only on historical trends of case reporting as input, this approach can be immediately adapted in a variety of disease settings.

Across diseases, NobBS outperformed the HH approach on accuracy of uncertainty estimates, and produced comparable or better point estimates. For the subset of weeks in which both models could produce forecasts (weeks with at least one case initially reported), point estimates for NobBS were substantially more accurate than the HH model for dengue cases (rRMSE improved by 300%) and slightly less accurate for ILI cases (rRMSE decreased by 19%). However, analysis of the probability distributions of the nowcasts revealed a much more substantial difference; the average score for NobBS was approximately twice as high for dengue and more than 10 times as high for ILI cases (Table 1). This indicates that the NobBS approach assigned much higher probability to the actual outcome, even though point accuracy was somewhat lower for the ILI cases.

While utilizing a similar modeling structure for case reporting delays as the HH model[9], NobBS introduces a simple dependency between case counts over time; that is, changes in case counts between weeks are assumed to be related via a first-order random walk process on the logarithmic scale. This feature is critical in the context of infectious disease transmission, where the number of true infections in a given week mechanistically depends in part on the number of true infections in previous weeks due to the infectious process, whether the pathogen is transmitted directly or by vectors[18]. Hence, variations of autoregressive models are common in disease forecasting[19,20]. Indeed, a recently published study describes a similar approach to NobBS [16], proposing the same model structure (an adaptation of the historic chain-ladder approach) and similar priors (namely, a first-order random walk on the underlying distribution of cases), finding that this approach performed well for two diseases. Two key differences in model structure are that NobBS parameterizes the delay distribution as a Dirichlet as opposed to a random walk, to model the underlying delay distribution as a probability vector, and omits a model intercept. We also aimed to identify and deconstruct the features that contribute to good performance, comparing to a benchmark approach and testing the robustness of NobBS to multiple delay distributions, different moving window sizes, and accuracy metrics. Our results suggest that this approach is robust across disease settings, and offers deeper insights into why: importantly, the temporal dependency between cases in the evolution of an outbreak.

As a result, when reporting delays are time-varying, as is often the case in epidemics[17], we show that the NobBS approach is less accurate compared to its performance in a stable delay distribution, but still shows improvement over the HH approach likely because the NobBS approach is informed by the number of cases experienced in previous weeks, not just the delay distribution, making it more robust to larger fluctuations. As mentioned, the HH approach software includes the option to model a time-varying delay explicitly in the nowcast approach, but requires specifying the time at which the delay distribution is expected to change (the change-point), which is generally not known in real-time. In addition, the authors of the HH approach introduce a feature to model the epidemic curve smoothly, however the implementation is bundled with the change-point model, and is therefore not possible to implement without the change-point component. In the case of the ILI and dengue data, there was no reason to believe reporting changed systematically at any point during the years covered by the data used here, and S4 Fig illustrates that a change-point model would likely be inappropriate given the irregular fluctuations in the delay probabilities over time. However, both features may improve performance of the HH model depending on the context, in particular when facing time-varying delays.

We also found that the accuracy of predictions is related at least in part to the number of cases reported to the surveillance system in week 0. When a larger proportion of cases were reported with no delay, as was the case for ILI compared to dengue, the point estimate accuracy was higher. This is not surprising, as a large fraction of true cases reported initially leaves fewer cases left to predict.

Further, we observed greater volatility in the nowcasts when the initial number of cases reported increases suddenly from low values. Two weeks in the dengue time series highlight this: August 3, 1998 and August 16, 2010. In those weeks, the number of cases initially increased by 16 and 17, respectively, from the previous week after 10 week with an average absolute change of 2.6 and 1.8 cases, respectively. Because this increase is an outlier in the distribution of reporting delays, in particular for delay *d* = 0, the model substantially overestimated the true number of cases before correcting the following week. We observed that shorter moving windows either exacerbated this issue (e.g. in 2010) or produced a similar overestimate (e.g. in 1998) (S7 Fig), which appears to be a consequence of the volatility in estimating the variance of the random walk process, despite more accurate estimation of the reporting delay probability (S8 Fig). While the smooth, autocorrelated relationship fit in the NobBS model helps reduce the effect of week-to-week variability in early reporting, it remains a challenge. Users should keep in mind these trade-offs when seeking to apply NobBS to their data.

While NobBS mitigates the effects of a time-varying delay distribution on case estimation, i.e. that the history of cases is leveraged to anchor case estimates to recent values, it does not explicitly model temporal changes in that delay; in other words, the estimated probability of a case occurring with delay = *d* is assumed to apply to all reporting weeks in the moving window. Shorter moving windows can improve estimation of the delay in the presence of changes, but explicitly estimating changes in that distribution may be explored for additional robustness in the presence of systemic changes in reporting. For example, authors in[21] propose a smooth estimate of the time-varying reporting delay distribution using p-spline smoothing. Specifying a time-specific change has also been proposed as mentioned previously [9], but empirical identification of a change point in real-time may be challenging or impossible in the context of nowcasting. The challenge that remains in all described approaches is the ability of the model to pick up on changes in the delay distribution that occur quickly, in other words that may otherwise be smoothed out by splines and long moving windows.

Lastly, the NobBS approach requires a trade-off between flexibility and precision when specifying the moving window size. With a shorter moving window the model will be more sensitive to recent reporting delay data, that could otherwise be “smoothed out” by the additional historical data considered by longer moving windows. While a narrower window allows more flexibility capture time-changes in the delay distribution, estimates of the parameters governing the random walk will be more volatile, with fewer data points from which to be estimated. In our experiments, the combination of shorter moving windows with rapid changes in cases reported produced large estimated variances, which often decreased the accuracy of nowcasts. We note that the moving window size is ultimately a choice by the modeler and should reflect consideration of available data and the desired smoothness of trends to be estimated; we discuss more in *Materials and Methods*.

A strength of the NobBS approach is that it only requires historical data on case reporting, which makes it immediately adaptable in a variety of disease systems that may not have other real-time covariates to inform the nowcasting model (e.g. search engine queries, weather data). However, if these covariates are available, it would be possible to include them directly in the log-linear model (*Materials and Methods*) as additional predictors, as demonstrated by Bastos et al [16].

Beyond supporting real-time disease tracking by public health officials, NobBS can complement existing disease forecast efforts by providing more accurate nowcasts to forecasting teams in the place of real-time reporting underestimates. For example, teams participating in the Centers for Disease Control and Prevention Epidemic Prediction Initiative (https://predict.cdc.gov) challenges (e.g. FluSight) use initial surveillance data for forecasting because it is the most up-to-date data available[22]. NobBS can help account for later revisions to these data and therefore improve prospective estimates as well, though we note for FluSight this would require nowcasting not only the total number of cases each week, but also the number of patient visits to achieve the ILI% required for forecasts. To provide guidance in the practical implementation of NobBS, we outline considerations for prior choice, window choice, and other features in *Materials and Methods*.

We present an R package, “NobBS,” intended to provide easy and flexible implementation of this approach to a wide audience of public health officials and researchers. This package is available on CRAN (cran.r-project.org/web/packages/NobBS/index.html).

## Materials and methods

### Surveillance data

We collected data on approximately 53,000 cases of dengue in Puerto Rico and 2.77 million cases of ILI in the United States over a 21-year (1092 weeks) and 3.75-year (196 weeks) period, respectively. Time-stamped weekly dengue data for laboratory-confirmed cases of dengue in Puerto Rico were collected by the Puerto Rico Department of Health and Centers for Disease Control and Prevention. The times used for the analysis were the time of onset as reported by the reporting clinician and the time of laboratory report completion. ILI data originated from the U.S. Outpatient Influenza-like Illness Surveillance Network (ILINet), which consolidates information from over 2000 outpatient healthcare providers in the United States who report to the CDC on the number of patients with ILI. The times used for the analysis were the week of ILI-related care seeking and the week when those cases were posted online in FluView (https://gis.cdc.gov/grasp/fluview/fluportaldashboard.html) as collected in the DELPHI epidemiological data API (https://github.com/cmu-delphi/delphi-epidata). ILI data with delays of more than 6 months occasionally had irregularities, so we restricted the analyses to delays of up to 6 months.

### Reporting triangle

Delays in reporting are often structurally decomposed into a (*T* x *D*) dimensional “reporting triangle,” where *T* is the most recent week (“now”) and *D* is the maximum reporting delay, in weeks, observed in the data. The data are right-truncated, since at any given week *t*, delays longer than *T–t* cannot be observed. For example, at week *t = T*, only the cases reported with delay *d* = 0 are observable; cases reported with longer delays (i.e. 1- or 2-week delays, *d = 1* or *d = 2*) will be known in future weeks. In S6 Table, we present an example of the reporting triangle using ILI data.

For each week *t*, the goal of nowcasting is to produce estimates for the total number of cases eventually reported, *N*_*t*_, based on an incomplete set of observed cases with delay *d*, *n*_*t*,*d*_. Since not every *n*_*t*,*d*_ is observed for a delay *d*, but will be observed at some unknown time point in the future, *N*_*t*_ = sum(*n*_*t*,*d*_).

Our approach is motivated by modeling the marginal cell counts of the reporting triangle, *n*_*t*,*d*_ in an adaptation of the loglinear chain ladder method developed in actuarial literature[13,14].

### Bayesian nowcast model

Let *n*_*t*,*d*_ be the number of cases reported for week *t* with delay *d*. We assume that the underlying cases occur in a Poisson process such that

$$n_{t,d}∼Pois(\lambda_{t,d}).$$

We apply this default assumption in the case of the dengue fever data.

We also allow for extra-Poisson variation, that is, when the variance is larger than the mean and a negative binomial process (of which the Poisson is a special case) is more appropriate. We apply this in the case of the influenza (ILI) data:

$$n_{t,d}∼NB(r,p_{t,d})\;\mathrm{where}$$

$$p_{t,d}=r/(r+\lambda_{t,d}),$$

and p(t,d) is the failure probability at week *t* and delay *d*, and (r) is the number of failures before stopping.

We then model the mean, *λ*_*t*,*d*_, as a simple log-linear equation

$$log\;(\lambda_{t,d})=\alpha_{t}+{log(\beta }_{d}),$$

where *α*_*t*_ represents the true epidemiologic signal for week *t* and *β*_*d*_ as the probability of reporting with delay = *d*. In other words, NobBS contains random effects for week *t* and the reporting delay *d*. Exponentiating both sides of the equation, $\lambda_{t,d}=e^{\alpha_{t}}*\beta_{d}$. This model is an adaptation of the general chain ladder model, proposed in the actuarial literature as an approach to nowcast the number of claims at time *t* with delay *d* with the form *log* (*λ*_*t*,*d*_) = *μ*+*α*_*t*_+*β*_*d*_ [15].

We place prior distributions on *α*_*t*_ and *β*_*d*_ reflecting properties of each parameter. Since *β*_*d*_ represents a probability vector containing delays = 0, …, *D*, we place on it a Dirichlet prior of length *D*:

$$\beta_{d}∼Dir(\theta )$$

$$\theta =(\theta_{0},\ldots ,\theta_{D})$$

The maximum delay *D* can be identified as the maximum observable delay in the data, which may change as the time series extends, or can be fixed at some value *D* thought to represent a very long delay. In the latter case, *θ*_*D*_ can be modeled as the probability of delay ≥ *D*. For dengue, we choose to fix *D* at 10 weeks, since over 99% of the cases observed in the first two years (prior to producing out-of-sample nowcasts) were reported within 10 weeks. For influenza, we chose *D* to be the longest possible delay within the 27-week moving window, or *D* = 26. The implications of choosing a maximum delay *D* within a moving window of *W* weeks means that the nowcast will include all cases arising with delays greater than or equal to *D* but less than or equal to *W*, thus excluding all cases with delays greater than *W* (see the reporting triangle in S6 Table).

We place weakly informative priors on *θ* representing a small number of hypothetical total cases (n = 10) distributed across delay bins, loosely representing the empirical probability of reporting delays for each delay *d* observed in the first two years of data for dengue and the first 6 months of data for ILI (training periods). As a sensitivity, we also placed weak priors on *θ* treating all delays with equal probability, but there was no material difference in the results.

We allow a dependency between successive *α*_*t*_’s to capture the time evolution and autocorrelation of cases from week-to-week, commonly exhibited by epidemic curves. We therefore model *α*_*t*_ as a first-order random walk:

$$\alpha_{t=1}=N(0,0.001)$$

$$\alpha_{t>1}∼N(\alpha_{t-1},\tau_{\alpha }^{2})$$

Because *α*_*t*_ is in natural log form, this constitutes a geometric random walk.

We place weakly informative priors on the precisions of the Normal distribution, $\tau_{\alpha }^{2}∼Ga(0.01,0.01)$. For the negative binomial stopping-time parameter, r, we place an informative Ga(60,20) prior to reflect belief that the process deviates moderately from the Poisson.

Models were compiled in JAGS on R (v 3.3.2) producing 10,000 posterior samples. Trace plots were visually reviewed for convergence.

### NobBS R package implementation and recommendations

In the R package “NobBS” (cran.r-project.org/web/packages/NobBS/index.html), the nowcast model may be implemented automatically by specifying a data frame with one record (row) per case, with a variable specifying the date of case onset and a variable specifying the date of case report. Dates are assumed to be in a sequence of weeks or months, with no gaps in reporting (i.e. no weeks for which reporting did not happen). By default, the model runs a Poisson log-linear model with no moving window (i.e. all historical data is taken into account), a maximum delay *D* equal to 1 minus the length of the reporting time series (i.e. if 12 consecutive weeks of report data are provided, the longest possible delay is equal to 11 weeks), and ignoring any delays longer than *D* (because they are unobserved in the default implementation). By default, a weakly informative Ga(0.01,0.01) prior is placed on the precision of the first-order random walk, $\tau_{\alpha }^{2}$, indicating equal weight to a standard deviation of 1 as to a standard deviation of 10,000: in other words, little knowledge about the extent to which the number of cases in the present week differ from the previous week. A weakly informative Dirichlet(*θ*_*d*…*D*_ = 0.1) prior is placed on the vector of delay probabilities, *β*_*d*_, indicating the weak prior belief that the probability of delay *d* is equal at all delays.

However, in “NobBS” all of these features are customizable and it is possible to leverage existing knowledge or historical information to impose more informative priors on the data. For example, it may be already known in the surveillance field that cases of dengue fever are rarely reported the same week they occur (i.e. that the probability of delay *d* = 0 is very low), but that most cases are reported within 4 weeks. In this example, it would be possible to design a stronger prior on *β*_*d*_ such that *θ* = (3,30,50,10,4,3), indicating that out of 100 cases distributed across delays, 3% of cases are expected to be reported with no delay, 30% of cases with a 1-week delay, 50% of cases with a 2-week delay, 10% of cases with a 3-week delay, 4% of cases with a 4-week delay, and 3% of cases with a 5-week delay. In other words, the user may directly parameterize the Dirichlet prior using knowledge of the delay distribution, and the sum of *θ*_*d*…*D*_ is related to the strength of the prior. Historical information (e.g. reporting data from the last several months or years) may also be used to inform the Dirichlet prior, such as through plotting the empirical distribution of delays in case reports. In the present study, for example, the observed delay distribution over the initial model training period was used to inform weakly informative Dirichlet priors for dengue and ILI, with the sum of *θ*_*d*…*D*_ being small.

The shape and rate hyperpriors of the Gamma(shape, rate) prior on $\tau_{\alpha }^{2}$ may also be modified to reflect stronger prior knowledge of the relationship between cases from week-to-week, which controls the smoothness of the epidemic curve. Increasing the shape parameter with a more informative prior, e.g. Gamma(10,0.1), can force the differences in cases from week-to-week to be small, resulting in a smoother epidemic curve and mitigating large fluctuations in estimates.

In all cases, keep in mind that the posterior is a compromise between the prior and the likelihood, so with enough weeks of reporting, the observed data can dominate over the prior.

Specifying a moving window size (e.g. a moving window of 27 weeks) may be leveraged for computational advantage (faster runtime) or to reflect the belief that the delay distribution of very old reports is no longer relevant to inform current reports. Alternatively, the user may prefer to let the moving window expand each week, especially to leverage as much prior data as possible to inform the nowcasts in the absence of available historical information. As discussed in Results, the estimation of the parameters is sensitive to how much historical data is used to fit the model, and this decision is up to the user and could depend on the relevant disease reporting system. The moving window acts to consider (or “weight”) all past observations equally, and shortening the moving window reflects the extent to which the user wishes to consider only more recent data as informative–noting also that shorter moving windows produce more volatile estimates of delay probabilities (which generally increases the accuracy of) as well as more volatile estimates of the variance of the random walk (which generally decreases the accuracy of). Still, these parameters may be constrained through their priors as discussed above.

### Nowcast estimates

We produced weekly nowcasts beginning with the 27^th^ week (influenza) and 104^th^ week (dengue) and through the final week of the series. This resulted in 989 weekly out-of-sample estimates of dengue cases and 170 weekly out-of-sample estimates of ILI. The time series of nowcast estimates for both diseases are shown in Figs 1 and 2.

We used a two-year moving window to estimate a stable delay distribution within the window. As a sensitivity, and to gauge the minimum amount of historical information required to produce accurate nowcasts, we also applied moving windows of 5, 12, and 27 weeks (approximately 6 months).

We used as a benchmark for comparison the “nowcast” function of the R package “surveillance” by Höhle and colleagues (described in ref.[9]) designed to produce Bayesian nowcasts for epidemics using a hierarchical model for *n*_*t*, *d* ≤ *T-t*_ | *n*_*t*,*d*_, or the observed cases conditional on the expected total number of cases. We applied the function assuming a time-homogenous delay distribution and recommended parameterization described by the authors in http://staff.math.su.se/hoehle/blog/2016/07/19/nowCast.html, and for comparability, used the same moving window sizes (27 and 104 weeks) to produce nowcasts over the same time periods.

### Model performance metrics

The mean absolute error (MAE), root mean square error (RMSE) and relative root mean square error (rRMSE) are defined, respectively, as:

$$MAE=\frac{1}{n}\sum_{i=1}^{n}abs(y_{i}-x_{i})$$

$$RMSE=\sqrt{\frac{1}{n}\sum_{i=1}^{n}{\left(y_{i}-x_{i}\right)}^{2}}$$

$$rRMSE=\sqrt{\frac{1}{n}\sum_{i=1}^{n}{\left(\frac{y_{i}-x_{i}}{y_{i}}\right)}^{2}}$$

and were used to quantify the accuracy of point estimates, x_i_, compared to true case numbers, y_i_, across the different models at each week *i*.

To quantify the accuracy of the point estimates in capturing the *change* in cases from week *i-1* to week *i*, we computed the mean absolute error of the change (MAEΔ) and the RMSE of the change (RMSEΔ):

$$MAE\Delta =\frac{1}{n-1}\sum_{i=2}^{n}abs({(x}_{i}-x_{i-1})-(y_{i}-y_{i-1}))$$

$$RMSE\Delta =\sqrt{\frac{1}{n-1}\sum_{i=2}^{n}{\left((y_{i}-y_{i-1})-(x_{i}-x_{i-1})\right)}^{2}}$$

To capture smoothness in predictions from week-to-week, we also calculated the lag-1 autocorrelation of predictions (ρ_a_) and cases (ρ_c_) between week *i* and week *i-1*.

$$\rho =\frac{\sum_{i=2}^{n}(x_{i}-\bar{x})(x_{i-1}-\bar{x})}{\sum_{i=2}^{n}({x_{i}-\bar{x})}^{2}}$$

where x = the predicted or true cases at each week *i*.

The logarithmic scoring rule was used to quantify the accuracy of the posterior predictive distribution of the nowcast. Predictive distributions were assigned to a series of bins categorized across possible values of true case counts. We used bin widths of 25 cases for dengue and 1000 cases for influenza, allowing for a larger number of bins for ILI cases based on case ranges of approx. 0–400 for dengue and 4,000–40,000 for ILI. For a predictive distribution with binned probability pi for a given nowcast target, the logarithmic score was calculated as ln(pi). For example, there were 115 cases eventually observed for the week of January 20, 1992. The NobBS nowcast for this week, which assigned a probability of 0.4 to the bin [100,125), thus received a log score of ln(0.4) = -0.92. As in[22,23], a very low log score of -10 was assigned for weeks in which the predictive distribution did not include the true case value, for weeks in which the bin probability ≤ e-10. This rule provides a lower limit (-10) to the score of highly inaccurate predictions.

The average log score across all prediction weeks was computed for all models to assess nowcast performance. The exponentiated average log score yields a nowcast score that can be interpreted as the average probability assigned to the bin corresponding to the true number of cases, and is a metric for model comparison purposes used in several other forecast contexts[22,23]. In this paper, we present the exponentiated average log score and refer to this as the average score.

### Simulated ILI data

To simulate ILI data with a time-varying probability of reporting delay *d* = 0, we drew, for each week, Pr(d = 0) from Unif(0.2, 0.9) for all weeks in which the total number of eventually-observed cases exceeded the mean of the ILI series (14,000 cases), and from Unif(0, 0.65) for all weeks in which the total observed case count was less than or equal to 14,000. This probability was used to calculate the simulated number of cases that would be observed with d = 0, out of the total number of cases that would be eventually observed for that week. The remaining cases were distributed to other delays ranging from 1–52 weeks using NB(0.9,0.4). This produced a rough approximation for a hypothetical scenario in which cases are reported faster (higher probability of d = 0) during weeks with higher disease activity (more cases), representing heightened awareness of an outbreak and increased urgency of reporting. However, it would also be possible to simulate the reverse scenario (slower reporting at weeks of higher incidence, to demonstrate the problem of reporting backfill during an outbreak) by swapping the two uniform priors.

## Supporting information

S1 Table NobBS Average error for moderate and high absolute changes in initial case reports, compared to the previous week. (PDF)

S2 Table Comparing model performance on influenza reports with constant and non-constant delay distributions. (PDF)

S3 Table Performance measures for estimates of the change in ILI incidence from the previous week, comparing constant and non-constant ILI delay distributions. (PDF)

S4 Table Outbreak performance measures for each nowcast model, by disease. A dengue outbreak season was defined as weeks for which the number of cases exceeded 50, and an ILI outbreak season was defined per the CDC as Epidemiologic Weeks 40 through Week 20 of the following year.
(PDF)

S5 Table Select performance measures for dengue fever nowcast model with different moving window sizes. (PDF)

S6 Table Reporting triangle for ILI on June 8, 2015. The *TxD* reporting triangle decomposes the number of cases, n_t,d_, reported for each week *t* (rows) and each delay *d* (column), up to a delay of *D* = 26 weeks within the 27-week moving window. The goal of nowcasting is to predict the missing (NA) n_t,d_’s.
(PDF)

S1 Fig **Prediction interval calibration plot for ILI nowcasts showing the percent of true ILI cases contained in the prediction interval (y-axis) at deciles of the prediction interval (x-axis, 10% to 90%).** Perfect calibration is shown by the dotted blue line.
(PNG)

S2 Fig The delay distribution (grey) and cumulative distribution (red), in weeks, over the full time series for (A) dengue fever and (B) influenza-like illness (ILI) cases.
(PNG)

S3 Fig Comparing (A) the change in initial case reports (from previous week) to (B) the error of NobBS for dengue fever.
(PNG)

S4 Fig Weekly reporting delay probabilities for delays up to 17 weeks for (A) dengue fever from 1990–2010 and (B) influenza-like illness from 2014–2017.
(PDF)

S5 Fig Weekly ILI nowcasts for June 30, 2014 through March 14, 2016 using a non-constant (time-varying) delay distribution and 2-year moving window. (A) NobBS nowcasts along with (B) point estimate and uncertainty accuracy, as measured by the score and the prediction error, are compared to (C) nowcasts by the HH approach with (D) corresponding scores and prediction errors. For nowcasting, the number of newly-reported cases each week (blue line) are the only data available in real-time for that week, and help inform the estimate of the total number of cases that will be eventually reported (red line), shown with 95% prediction intervals (pink bands). For the HH approach, the 95% prediction intervals are very narrow and are thus difficult to see. The true number of cases eventually reported (black line) is known only in hindsight and is the nowcast target. The score (brown line) and the difference between the true and mean estimated number of cases (grey line) are shown as a function of time.
(PDF)

S6 Fig **Comparing the probability assigned to the bin containing the true number of cases (y-axis) to the true number of cases (x-axis), for weekly dengue fever nowcasts using NobBS.** Vertical dashed lines in grey are used to visualize the bin width of 25 cases.
(PNG)

S7 Fig **Weekly NobBS dengue fever nowcasts using (A) 5-week moving window, (B) 12-week moving window, and (C) 27-week (approx. 6 month) moving window.** Plots are zoomed in the y-axis to show the details of prediction.
(PNG)

S8 Fig **Comparing the (A) estimated reporting delay probabilities for delay *d* = 0 and (B) estimated inverse variance of the random walk, at moving windows of 5 weeks (blue) and 104 weeks (red) for dengue fever nowcasts.** In (A), the true reporting delay probability at *d* = 0 can be calculated from the data and is shown in grey.
(PNG)
