# Supplementary material for: Nowcasting by Bayesian Smoothing: A flexible, generalizable model for real-time epidemic tracking
Source: PLoS Comput Biol. 2020 Apr 6;16(4):e1007735. doi: 10.1371/journal.pcbi.1007735 (PMC7162546; doi:10.1371/journal.pcbi.1007735)
Supplement: S1 Table — (PDF) [file pcbi.1007735.s001.pdf]

		Average error (predicted - actual) for weeks where:	
**Disease**	**Threshold**	**Δ initial reports > threshold**	**Δ initial reports <= threshold**
Dengue	Moderate: 5 cases	61.6	-1.8
	High: 10 cases	277.0	-0.2
ILI	Moderate: 1,000 cases	-92.8	-38.4
	High: 2,500 cases	-198.9	-37.7
