# Supplementary material for: Nowcasting by Bayesian Smoothing: A flexible, generalizable model for real-time epidemic tracking
Source: PLoS Comput Biol. 2020 Apr 6;16(4):e1007735. doi: 10.1371/journal.pcbi.1007735 (PMC7162546; doi:10.1371/journal.pcbi.1007735)
Supplement: S2 Table — (PDF) [file pcbi.1007735.s002.pdf]

Model	Influenza: Constant delay						Influenza: Non-constant (time-varying) delay				
	Period	MAE	rRMSE	RMSE	Average Score	95% PI coverage	MAE	rRMSE	RMSE	Average Score	95% PI coverage
NobBS	06/30/2014	777.9	0.081	1135.2	0.172	1.00	3476.5	0.302	4622.7	0.06	0.93
HH (ref. 9)	/2014 - 03/1[illegible]	689.9	0.072	15559.2	0.016	0.00	7315	0.621	10300.4	8.71E-05	0.57
