# Supplementary material for: Nowcasting by Bayesian Smoothing: A flexible, generalizable model for real-time epidemic tracking
Source: PLoS Comput Biol. 2020 Apr 6;16(4):e1007735. doi: 10.1371/journal.pcbi.1007735 (PMC7162546; doi:10.1371/journal.pcbi.1007735)
Supplement: S3 Table — (PDF) [file pcbi.1007735.s003.pdf]

Model	Period	Influenza: Constant delay				Influenza: Non-constant (time-varying) delay			
		MAEΔ	RMSEΔ	ρ_a	RMAΔ	MAEΔ	RMSEΔ	ρ_a	RMAΔ
NobBS	06/30/2014 - 03/14/2016	804	1268.3	0.96	1.01	4519.573	6745	0.78	3.77
HH (ref. 9)	06/30/2014 - 03/14/2016	758	1252.7	0.96	1.08	9437.18	14169	0.60	7.63
