# Supplementary material for: Nowcasting by Bayesian Smoothing: A flexible, generalizable model for real-time epidemic tracking
Source: PLoS Comput Biol. 2020 Apr 6;16(4):e1007735. doi: 10.1371/journal.pcbi.1007735 (PMC7162546; doi:10.1371/journal.pcbi.1007735)
Supplement: S4 Table — A dengue outbreak season was defined as weeks for which the number of cases exceeded 50, and an ILI outbreak season was defined per the CDC as Epidemiologic Weeks 40 through Week 20 of the following year. (PDF) [file pcbi.1007735.s004.pdf]

Disease	High incidence* weeks only	Cases	NobBS				HH (ref. 9)			
			MAE	rRMSE	RMSE	Average Score	MAE	rRMSE	RMSE	Average Score
Dengue	1992	2986	18	0.278	22.8	0.186	33	0.509	38.1	0.111
	1993	950	15	0.278	18.4	0.246	27	0.496	31.7	0.130
	1994	4758	43	0.358	58.7	0.000	62	0.490	73.3	0.081
	1995	1073	21	0.370	23.6	0.174	30	0.473	40.0	0.137
	1996	1134	13	0.256	15.7	0.289	23	0.471	27.8	0.176
	1997	1732	20	0.309	23.6	0.213	26	0.363	32.8	0.160
	1998	5305	32	0.372	47.7	0.134	67	0.809	90.9	0.070
	1999	743	13	0.265	15.9	0.356	24	0.497	30.7	0.186
	2000	207	13	0.303	15.8	0.151	26	0.535	28.2	0.118
	2001	1525	21	0.254	24.4	0.257	45	0.567	54.5	0.090
	2003	544	10	0.254	15.5	0.419	54	1.175	70.7	0.095
	2005	1982	27	0.276	33.7	0.177	43	0.435	50.5	0.107
	2007	2596	74	0.546	92.8	0.000	112	0.725	137.4	0.006
	2008	168	29	0.527	29.5	0.059	83	1.482	83.0	0.103
	2009	1567	24	0.374	30.1	0.153	39	0.517	46.9	0.120
	2010	6602	78	0.603	139.8	0.000	133	0.895	208.7	0.030
Influenza	2014-2015	675744	969	0.063	1482.2	0.129	885	0.055	1401.8	0.016
	2015-2016	551267	982	0.086	1158.8	0.148	602	0.055	828.8	0.010
	2016-2017	713272	735	0.048	906.4	0.176	815	0.043	1044.8	0.005

* Dengue outbreak weeks are defined as weeks for which dengue cases exceeded 50.
Influenza outbreak weeks are defined per the CDC definition, as Epidemiologic Weeks 40 through Week 20 of the following year, per CDC standard.
