# Supplementary material for: Nowcasting by Bayesian Smoothing: A flexible, generalizable model for real-time epidemic tracking
Source: PLoS Comput Biol. 2020 Apr 6;16(4):e1007735. doi: 10.1371/journal.pcbi.1007735 (PMC7162546; doi:10.1371/journal.pcbi.1007735)
Supplement: S5 Table — (PDF) [file pcbi.1007735.s005.pdf]

Model	Moving window size	rRMSE	Average Score	Correlation
NobBS	5 weeks	7.381	0.368	0.275
	12	0.634	0.370	0.760
	27 weeks (approx. 6 months)	0.655	0.369	0.806
	104 weeks (approx. 2 years)	0.600	0.349	0.84
