# Supplementary material for: Nowcasting by Bayesian Smoothing: A flexible, generalizable model for real-time epidemic tracking
Source: PLoS Comput Biol. 2020 Apr 6;16(4):e1007735. doi: 10.1371/journal.pcbi.1007735 (PMC7162546; doi:10.1371/journal.pcbi.1007735)
Supplement: S6 Table — The TxD reporting triangle decomposes the number of cases, nt,d, reported for each week t (rows) and each delay d (column), up to a delay of D = 26 weeks within the 27-week moving window. The goal of nowcasting is to predict the missing (NA) nt,d’s. (PDF) [file pcbi.1007735.s006.pdf]

Delay *d* (weeks)

**Week *t***	**0**	**1**	**2**	**3**	**4**	**5**	**6**	**7**	**8**	**9**	**10**	**11**	**12**	**13**	**14**	**15**	**16**	**17**	**18**	**19**	**20**	**21**	**22**	**23**	**24**	**25**	**26**
**12/8/2014**	27487	0	741	504	105	207	40	75	15	24	13	213	6	66	20	6	0	2	0	2	2	0	0	0	0	0	0
**12/15/2014**	28964	4943	1963	601	232	73	101	94	32	105	372	11	18	18	5	0	0	0	2	25	0	0	13	0	0	0	NA
**12/22/2014**	32022	5648	1545	298	51	90	31	22	138	398	12	6	13	9	15	0	0	5	23	0	0	8	0	0	0	NA	NA
**12/29/2014**	32726	2372	931	75	236	170	19	141	616	0	5	15	5	4	9	0	2	0	0	1	7	0	0	0	NA	NA	NA
**1/5/2015**	27209	2608	718	330	151	64	184	105	72	42	16	60	11	11	0	11	0	6	4	0	0	7	1	NA	NA	NA	NA
**1/12/2015**	25789	3119	907	390	115	127	454	56	25	22	119	12	12	22	10	28	8	2	0	0	9	0	NA	NA	NA	NA	NA
**1/19/2015**	28531	2502	726	315	250	183	472	24	65	81	15	19	14	8	8	8	0	0	0	6	0	NA	NA	NA	NA	NA	NA
**1/26/2015**	25586	3157	519	263	256	499	54	111	121	40	15	7	8	1	9	1	1	18	14	2	NA	NA	NA	NA	NA	NA	NA
**2/2/2015**	23610	2348	718	465	710	62	145	90	55	25	1	13	0	5	0	0	3	48	0	NA	NA	NA	NA	NA	NA	NA	NA
**2/9/2015**	20391	2808	454	384	490	429	88	51	43	27	41	18	3	3	0	0	18	0	NA	NA	NA	NA	NA	NA	NA	NA	NA
**2/16/2015**	18139	1442	690	451	194	128	71	0	60	29	0	21	2	0	3	21	0	NA	NA	NA	NA	NA	NA	NA	NA	NA	NA
**2/23/2015**	15911	1955	504	306	340	104	7	4	78	31	15	8	0	23	3	0	NA	NA	NA	NA	NA	NA	NA	NA	NA	NA	NA
**3/2/2015**	15294	2037	345	528	103	18	16	163	1	7	5	1	14	11	3	NA	NA	NA	NA	NA	NA	NA	NA	NA	NA	NA	NA
**3/9/2015**	14863	1574	659	151	361	43	24	91	11	3	2	11	18	1	NA	NA	NA	NA	NA	NA	NA	NA	NA	NA	NA	NA	NA
**3/16/2015**	13708	2110	171	441	90	56	111	47	0	1	2	41	43	NA	NA	NA	NA	NA	NA	NA	NA	NA	NA	NA	NA	NA	NA
**3/23/2015**	13772	1372	606	76	186	73	115	0	0	0	8	39	NA	NA	NA	NA	NA	NA	NA	NA	NA	NA	NA	NA	NA	NA	NA
**3/30/2015**	12147	1886	275	252	95	159	62	23	2	16	47	NA	NA	NA	NA	NA	NA	NA	NA	NA	NA	NA	NA	NA	NA	NA	NA
**4/6/2015**	11688	1178	387	111	124	57	18	49	68	37	NA	NA	NA	NA	NA	NA	NA	NA	NA	NA	NA	NA	NA	NA	NA	NA	NA
**4/13/2015**	9612	1158	291	159	164	23	43	44	40	NA	NA	NA	NA	NA	NA	NA	NA	NA	NA	NA	NA	NA	NA	NA	NA	NA	NA
**4/20/2015**	9092	1161	208	136	6	31	30	29	NA	NA	NA	NA	NA	NA	NA	NA	NA	NA	NA	NA	NA	NA	NA	NA	NA	NA	NA
**4/27/2015**	9060	993	140	82	172	39	36	NA	NA	NA	NA	NA	NA	NA	NA	NA	NA	NA	NA	NA	NA	NA	NA	NA	NA	NA	NA
**5/4/2015**	8702	924	226	213	45	35	NA	NA	NA	NA	NA	NA	NA	NA	NA	NA	NA	NA	NA	NA	NA	NA	NA	NA	NA	NA	NA
**5/11/2015**	7558	1182	241	131	37	NA	NA	NA	NA	NA	NA	NA	NA	NA	NA	NA	NA	NA	NA	NA	NA	NA	NA	NA	NA	NA	NA
**5/18/2015**	7015	1311	482	46	NA	NA	NA	NA	NA	NA	NA	NA	NA	NA	NA	NA	NA	NA	NA	NA	NA	NA	NA	NA	NA	NA	NA
**5/25/2015**	6934	799	92	NA	NA	NA	NA	NA	NA	NA	NA	NA	NA	NA	NA	NA	NA	NA	NA	NA	NA	NA	NA	NA	NA	NA	NA
**6/1/2015**	5802	642	NA	NA	NA	NA	NA	NA	NA	NA	NA	NA	NA	NA	NA	NA	NA	NA	NA	NA	NA	NA	NA	NA	NA	NA	NA
**6/8/2015**	4708	NA	NA	NA	NA	NA	NA	NA	NA	NA	NA	NA	NA	NA	NA	NA	NA	NA	NA	NA	NA	NA	NA	NA	NA	NA	NA
