# Supplementary figures and images for: Nowcasting by Bayesian Smoothing: A flexible, generalizable model for real-time epidemic tracking
Source: PLoS Comput Biol. 2020 Apr 6;16(4):e1007735. doi: 10.1371/journal.pcbi.1007735 (PMC7162546; doi:10.1371/journal.pcbi.1007735)

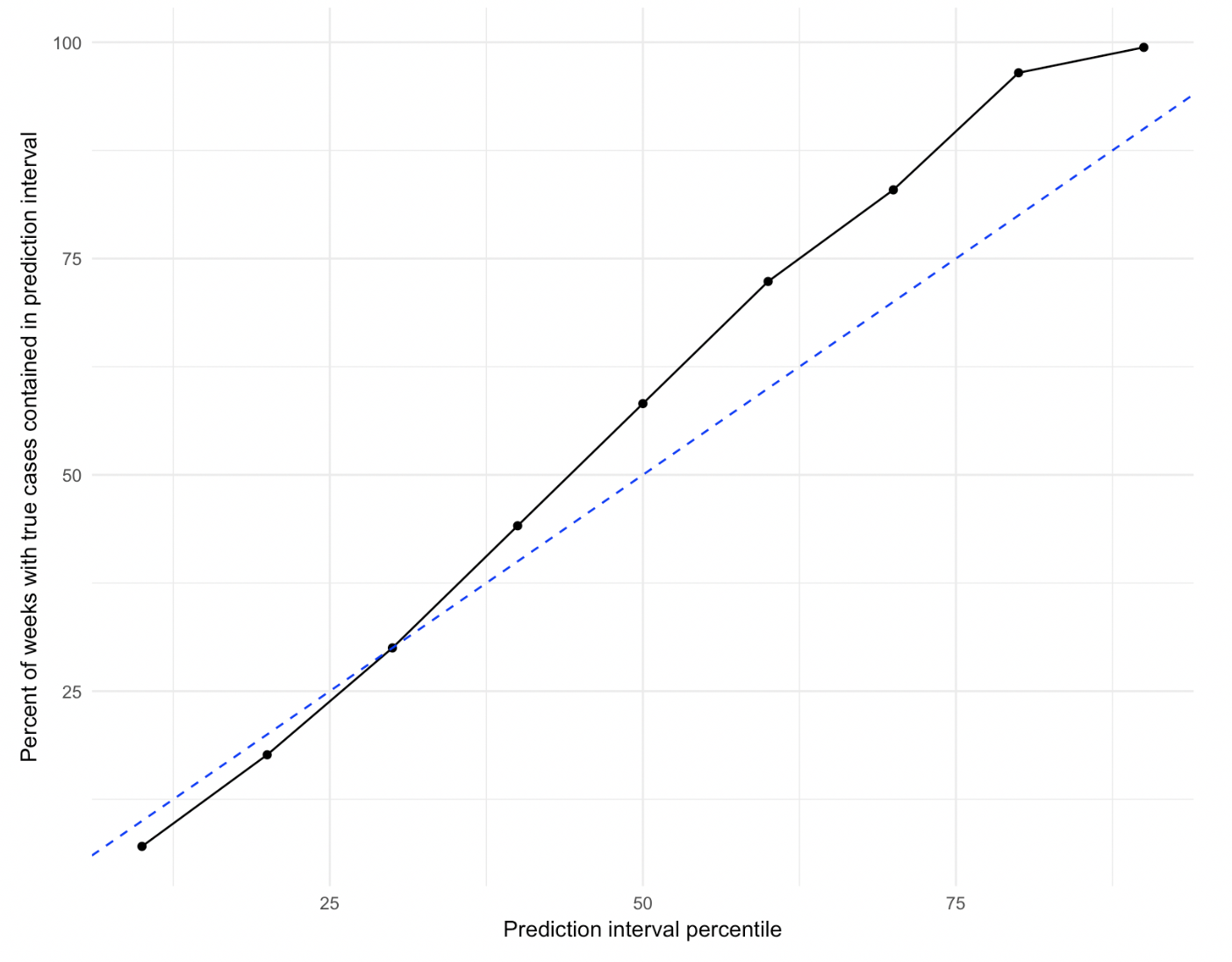

Supplement: S1 Fig — Prediction interval calibration plot for ILI nowcasts showing the percent of true ILI cases contained in the prediction interval (y-axis) at deciles of the prediction interval (x-axis, 10% to 90%). Perfect calibration is shown by the dotted blue line. (PNG) [file pcbi.1007735.s007.png]

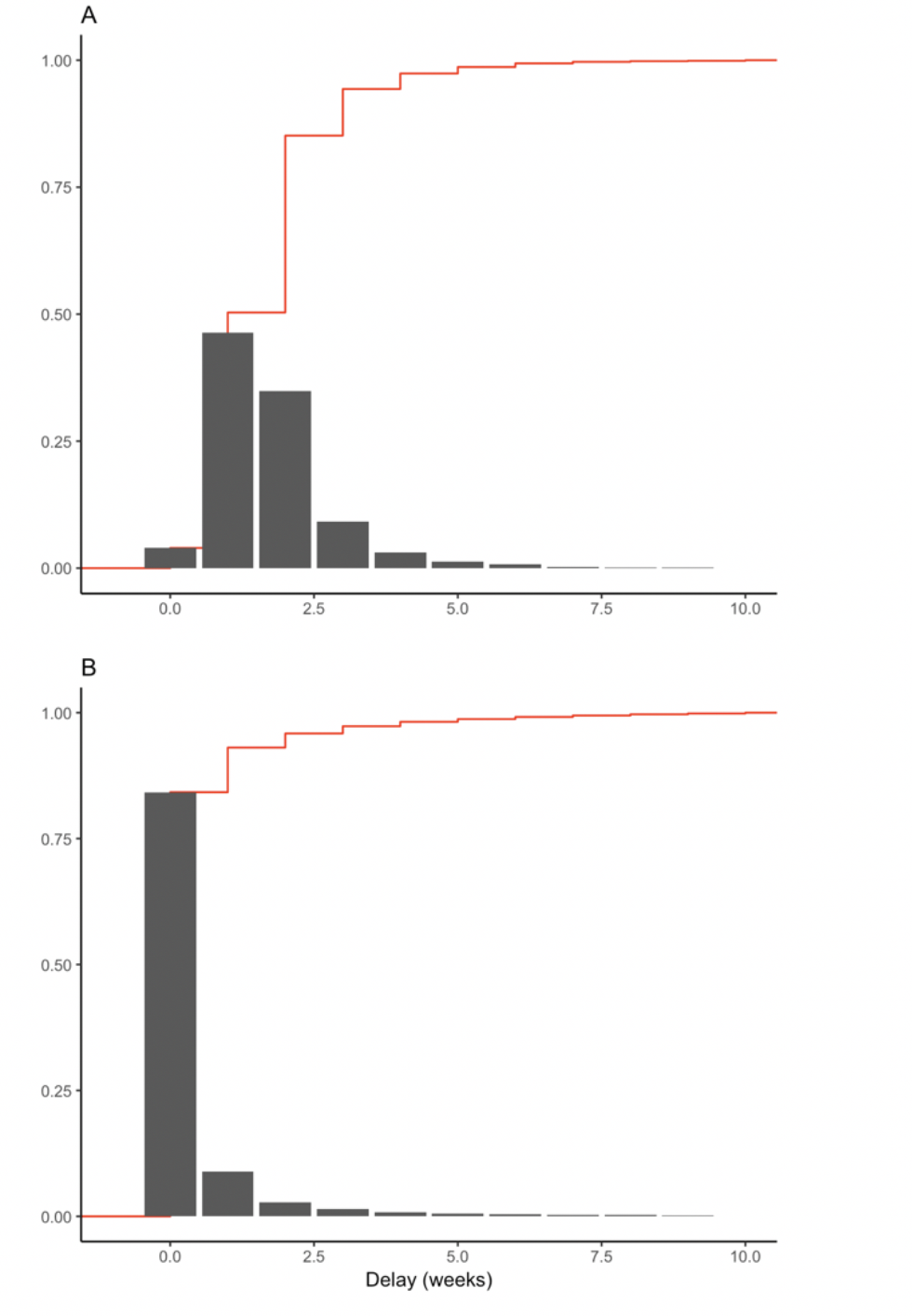

Supplement: S2 Fig — The delay distribution (grey) and cumulative distribution (red), in weeks, over the full time series for (A) dengue fever and (B) influenza-like illness (ILI) cases. (PNG) [file pcbi.1007735.s008.png]

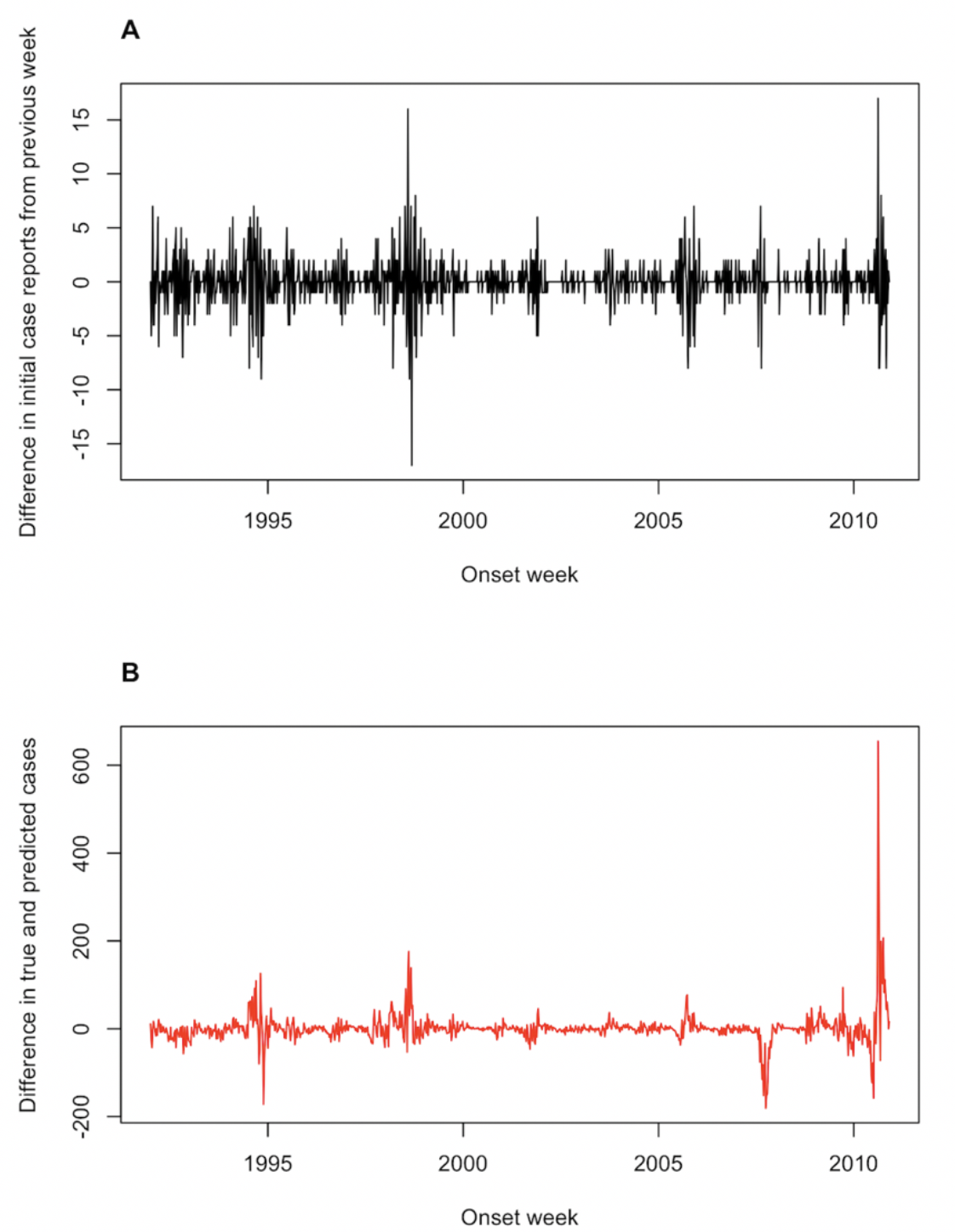

Supplement: S3 Fig — Comparing (A) the change in initial case reports (from previous week) to (B) the error of NobBS for dengue fever. (PNG) [file pcbi.1007735.s009.png]

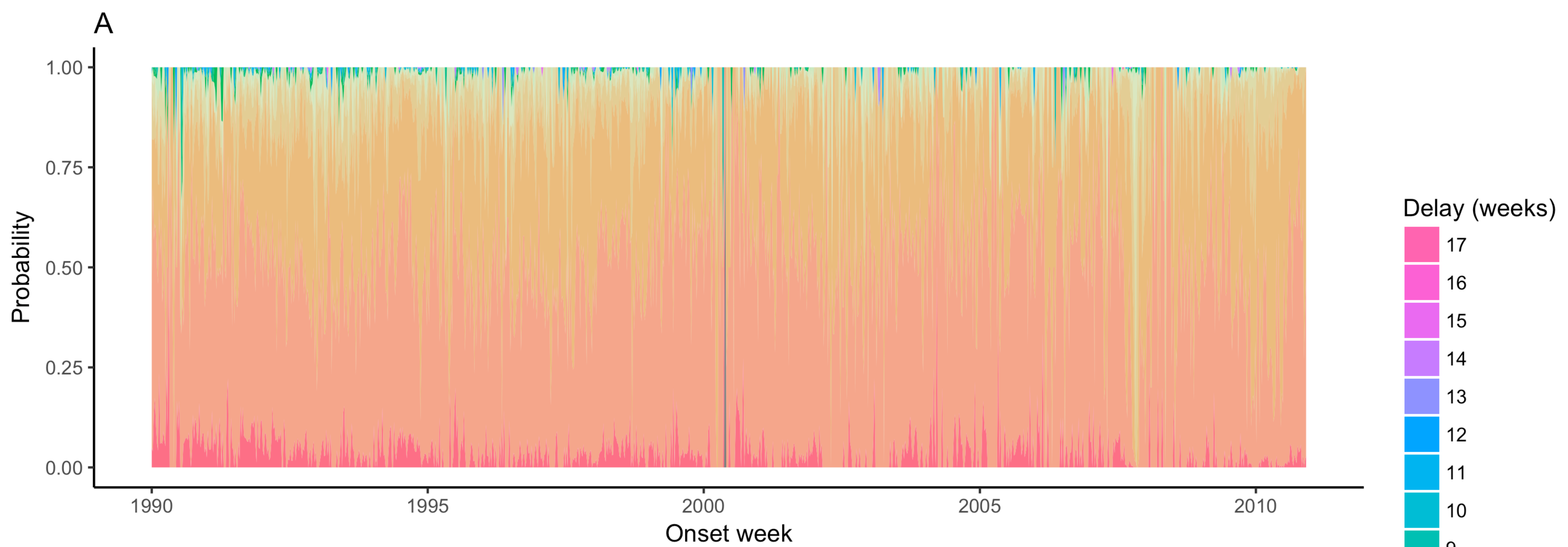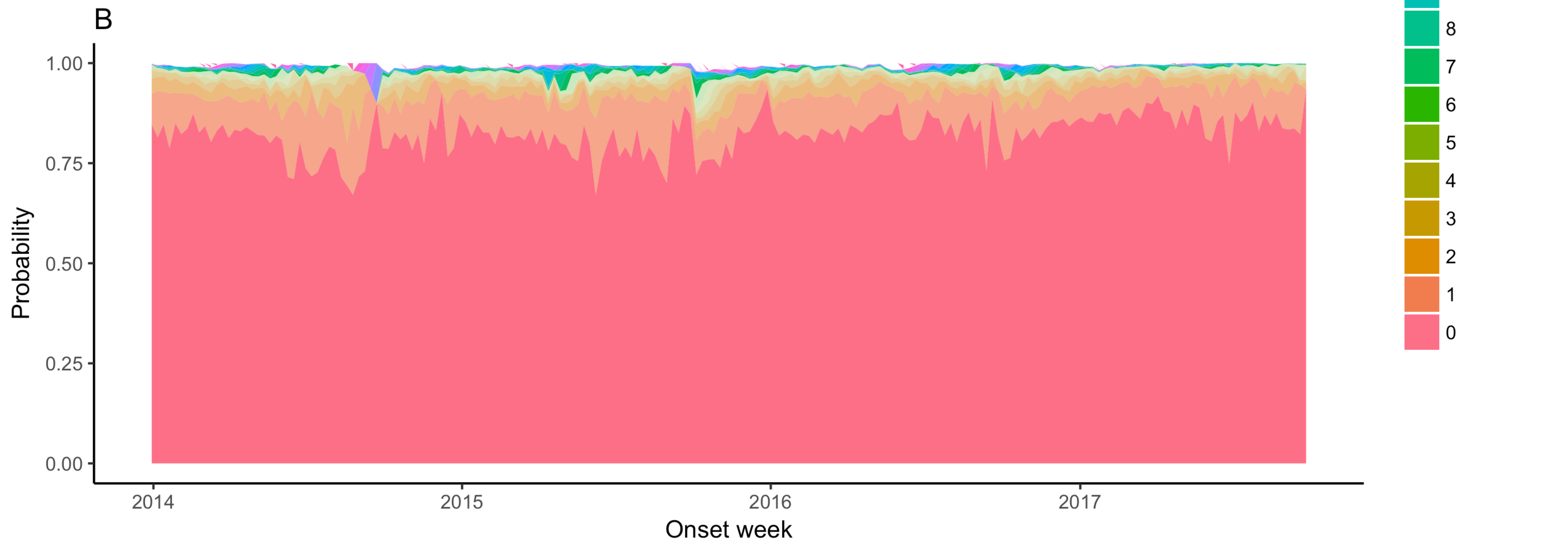

Supplement: S4 Fig — Weekly reporting delay probabilities for delays up to 17 weeks for (A) dengue fever from 1990–2010 and (B) influenza-like illness from 2014–2017. (PDF) [file pcbi.1007735.s010.pdf]

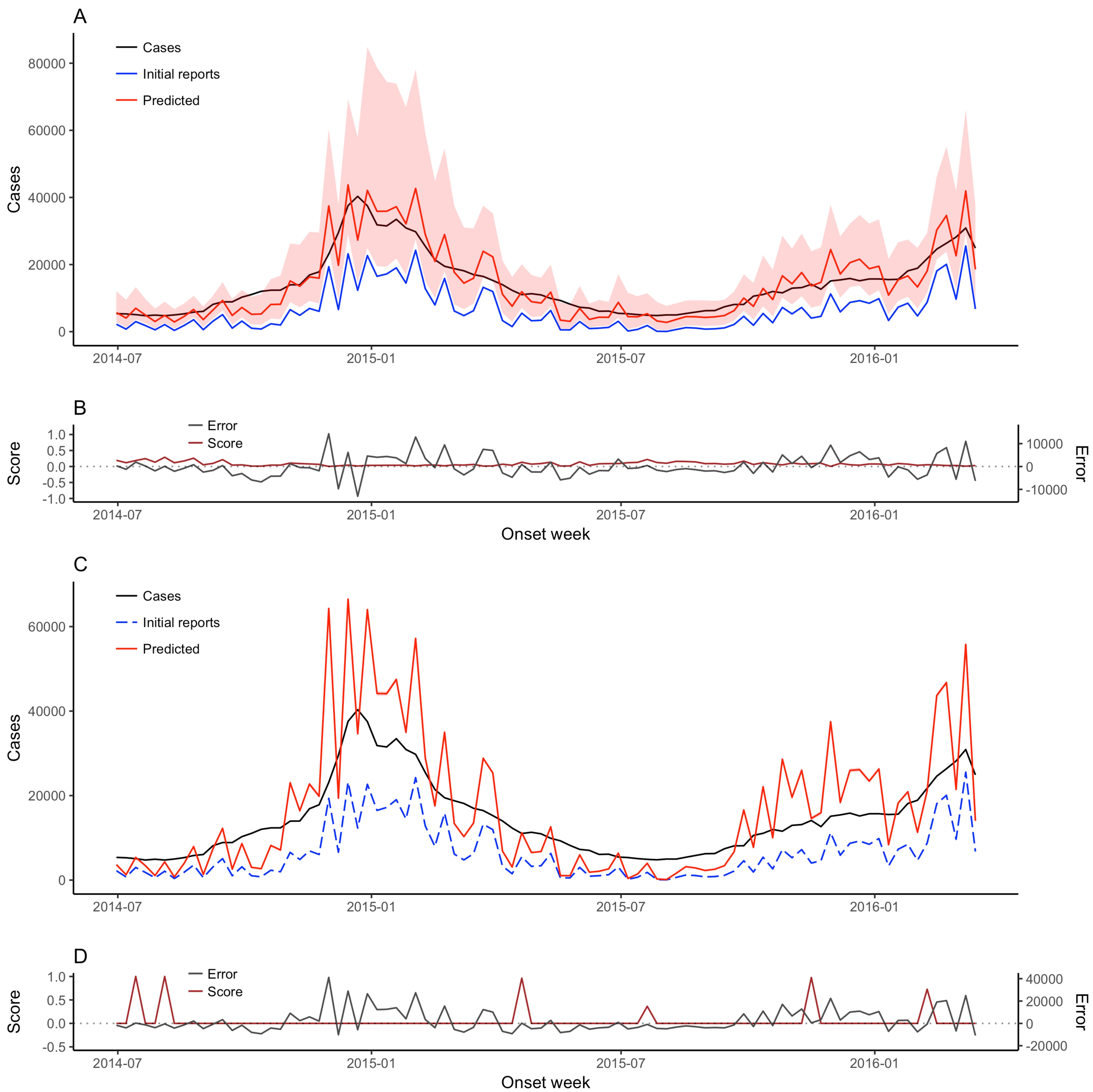

Supplement: S5 Fig — (A) NobBS nowcasts along with (B) point estimate and uncertainty accuracy, as measured by the score and the prediction error, are compared to (C) nowcasts by the HH approach with (D) corresponding scores and prediction errors. For nowcasting, the number of newly-reported cases each week (blue line) are the only data available in real-time for that week, and help inform the estimate of the total number of cases that will be eventually reported (red line), shown with 95% prediction intervals (pink bands). For the HH approach, the 95% prediction intervals are very narrow and are thus difficult to see. The true number of cases eventually reported (black line) is known only in hindsight and is the nowcast target. The score (brown line) and the difference between the true and mean estimated number of cases (grey line) are shown as a function of time. (PDF) [file pcbi.1007735.s011.pdf]

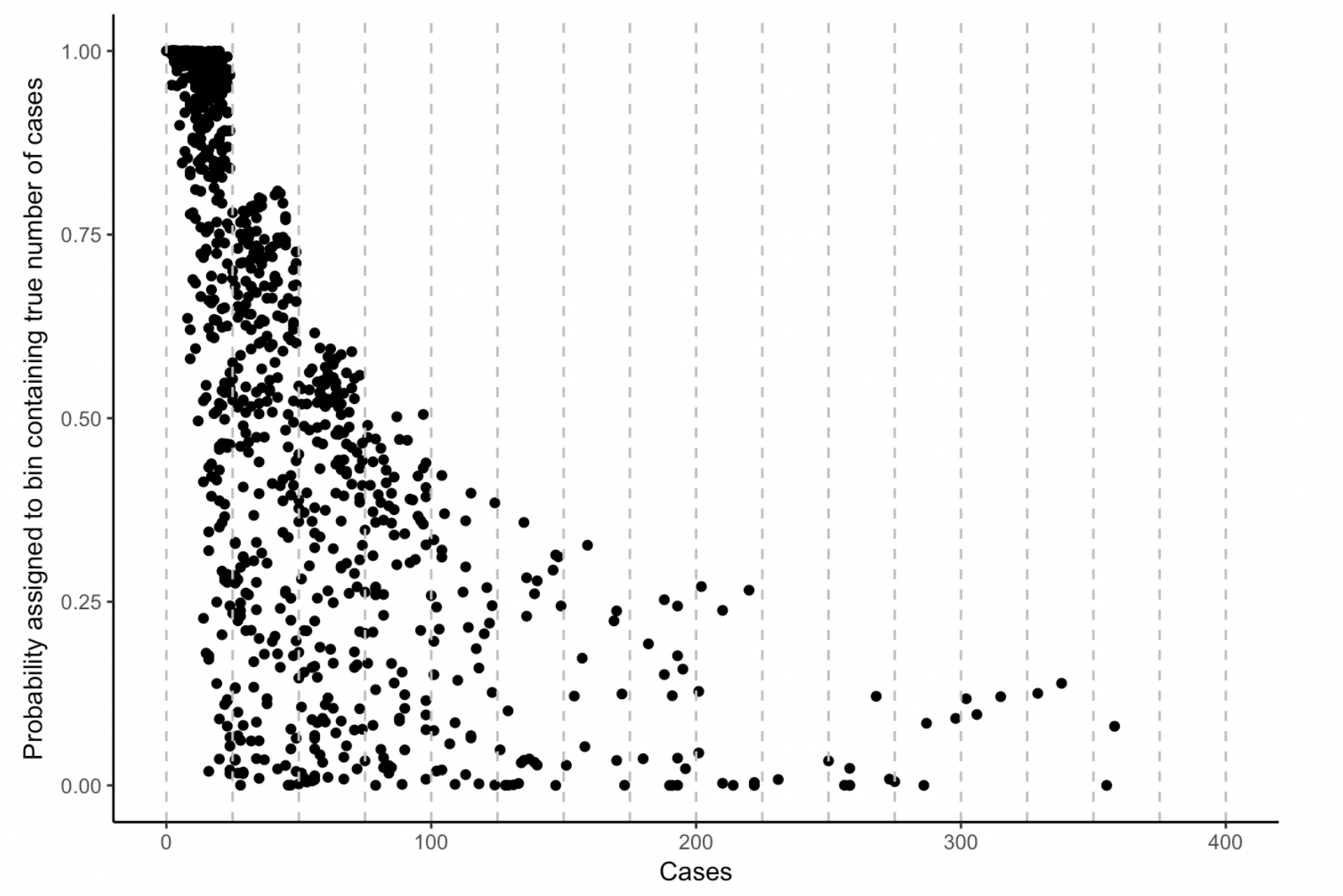

Supplement: S6 Fig — Comparing the probability assigned to the bin containing the true number of cases (y-axis) to the true number of cases (x-axis), for weekly dengue fever nowcasts using NobBS. Vertical dashed lines in grey are used to visualize the bin width of 25 cases. (PNG) [file pcbi.1007735.s012.png]

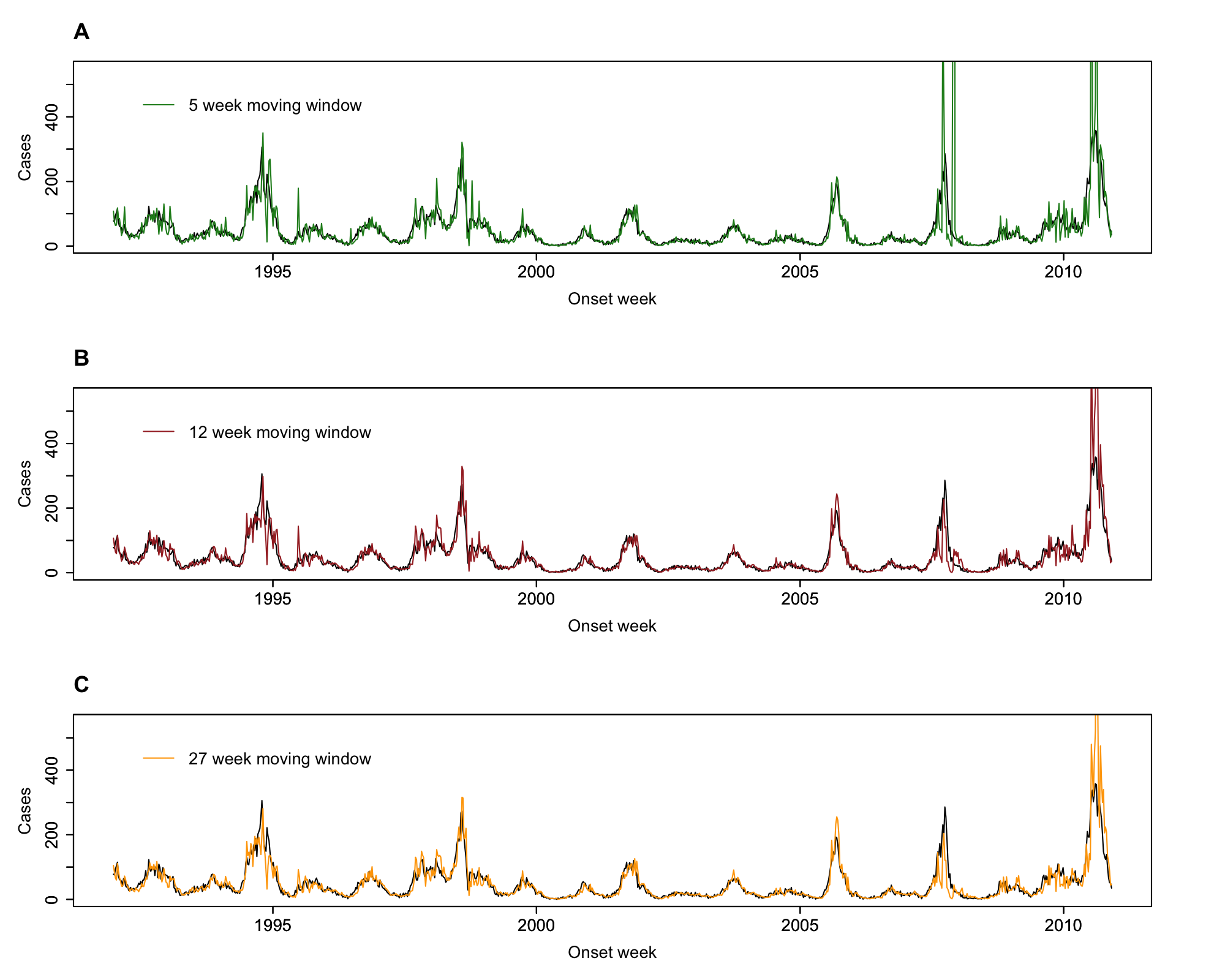

Supplement: S7 Fig — Weekly NobBS dengue fever nowcasts using (A) 5-week moving window, (B) 12-week moving window, and (C) 27-week (approx. 6 month) moving window. Plots are zoomed in the y-axis to show the details of prediction. (PNG) [file pcbi.1007735.s013.png]

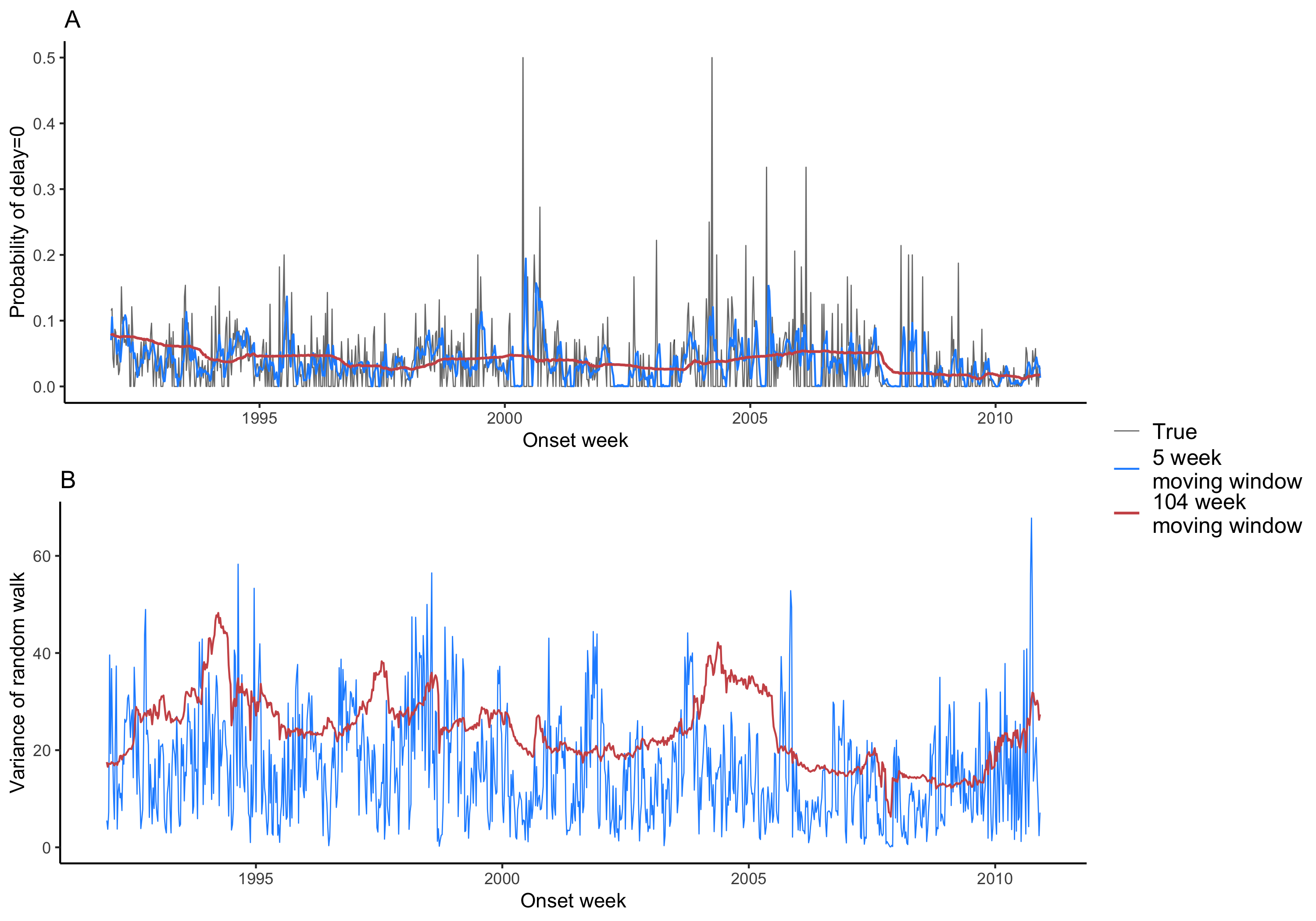

Supplement: S8 Fig — Comparing the (A) estimated reporting delay probabilities for delay d = 0 and (B) estimated inverse variance of the random walk, at moving windows of 5 weeks (blue) and 104 weeks (red) for dengue fever nowcasts. In (A), the true reporting delay probability at d = 0 can be calculated from the data and is shown in grey. (PNG) [file pcbi.1007735.s014.png]
